# Screening data from 19 patients with late‐onset Pompe disease for a phase I clinical trial of AAV8 vector‐mediated gene therapy

**DOI:** 10.1002/jmd2.12391

**Published:** 2023-08-17

**Authors:** William B. Hannah, Laura E. Case, Edward C. Smith, Crista Walters, Deeksha Bali, Priya S. Kishnani, Dwight D. Koeberl

**Affiliations:** ^1^ Division of Medical Genetics, Department of Pediatrics Duke University School of Medicine Durham North Carolina USA; ^2^ Doctor of Physical Therapy Division, Department of Orthopedics Duke University School of Medicine Durham North Carolina USA; ^3^ Division of Neurology, Department of Pediatrics Duke University School of Medicine Durham North Carolina USA

**Keywords:** adeno‐associated virus, gene therapy, Pompe disease

## Abstract

Late‐onset Pompe disease (LOPD) is a multisystem disorder with significant myopathy. The standard treatment is enzyme replacement therapy (ERT), a therapy that is lifesaving, yet with limitations. Clinical trials have emerged for other potential treatment options, including adeno‐associated virus (AAV) gene therapy. We present clinical parameters and AAV antibody titers for 19 individuals with LOPD undergoing screening for a Phase I clinical trial with an AAV serotype 8 vector targeting hepatic transduction (AAV2/8‐LSPhGAA). Reported clinical parameters included *GAA* genotype, assessments of muscle function, upright and supine spirometry, anti‐recombinant human GAA antibody titers, and biomarkers. Variability in measured parameters and phenotypes of screened individuals was evident. Eligibility criteria required that all participants have six‐minute walk test (6MWT) and upright forced vital capacity (FVC) below the expected range for normal individuals, and were stably treated with ERT for >2 years. All participants had Pompe disease diagnosed by enzyme deficiency, and all had the common c.‐32‐13T>G LOPD pathogenic variant. Screening identified 14 patients (74%) with no or minimal detectable neutralizing antibodies against AAV8 (titer ≤1:5). 6MWT distance varied significantly (percent of expected distance ranging from 24% to 91% with an average of 60 and standard deviation of 21). Upright FVC percent predicted ranged from 35% predicted to 91% predicted with an average of 66 and standard deviation of 18. None of the participants had significantly elevated alanine transaminase, which has been associated with LOPD and could complicate screening for hepatitis related to AAV gene therapy. We review the parameters considered in screening for eligibility for a clinical trial of AAV8 vector‐mediated gene therapy.

**Synopsis**

Screening data for a LOPD gene therapy trial provide insight into important considerations for enrollment criteria and surveillance of possible hepatitis. Among patients with LOPD screened for a phase I AAV gene therapy trial, 74% had no or minimal detectable pre‐existing antibodies to the AAV vector.

## 1 INTRODUCTION

Pompe disease is caused by deficiency of the enzyme acid alpha‐glucosidase (GAA) due to the recessive inheritance of biallelic disease‐causing variants in *GAA*. Late‐onset Pompe disease (LOPD) is distinguished from infantile‐onset Pompe disease (IOPD) by the absence of hypertrophic cardiomyopathy in the first year of life. Patients can present as early as the first year to later in life including adulthood.^1^, ^2^ LOPD is associated with variable expressivity with a broad phenotypic spectrum.^3^, ^4^, ^5^, ^6^, ^7^, ^8^, ^9^, ^10^, ^11^, ^12^, ^13^

Recombinant human GAA (rhGAA) enzyme replacement therapy (ERT) is standard of care treatment in individuals with LOPD. Infusion reactions and, rarely, anaphylaxis may occur. Some individuals receiving ERT develop IgG antibodies to the recombinant protein, limiting efficacy. Chronic disease sequelae persist in affected individuals with both LOPD and IOPD despite the use of ERT. Note, for instance, individuals with IOPD who have high and sustained antibody titers appear to have suboptimal ERT response.^14^ In short, despite the benefits of ERT, there are therapeutic limitations and concurrent therapies may be of benefit.

**FIGURE 1:**
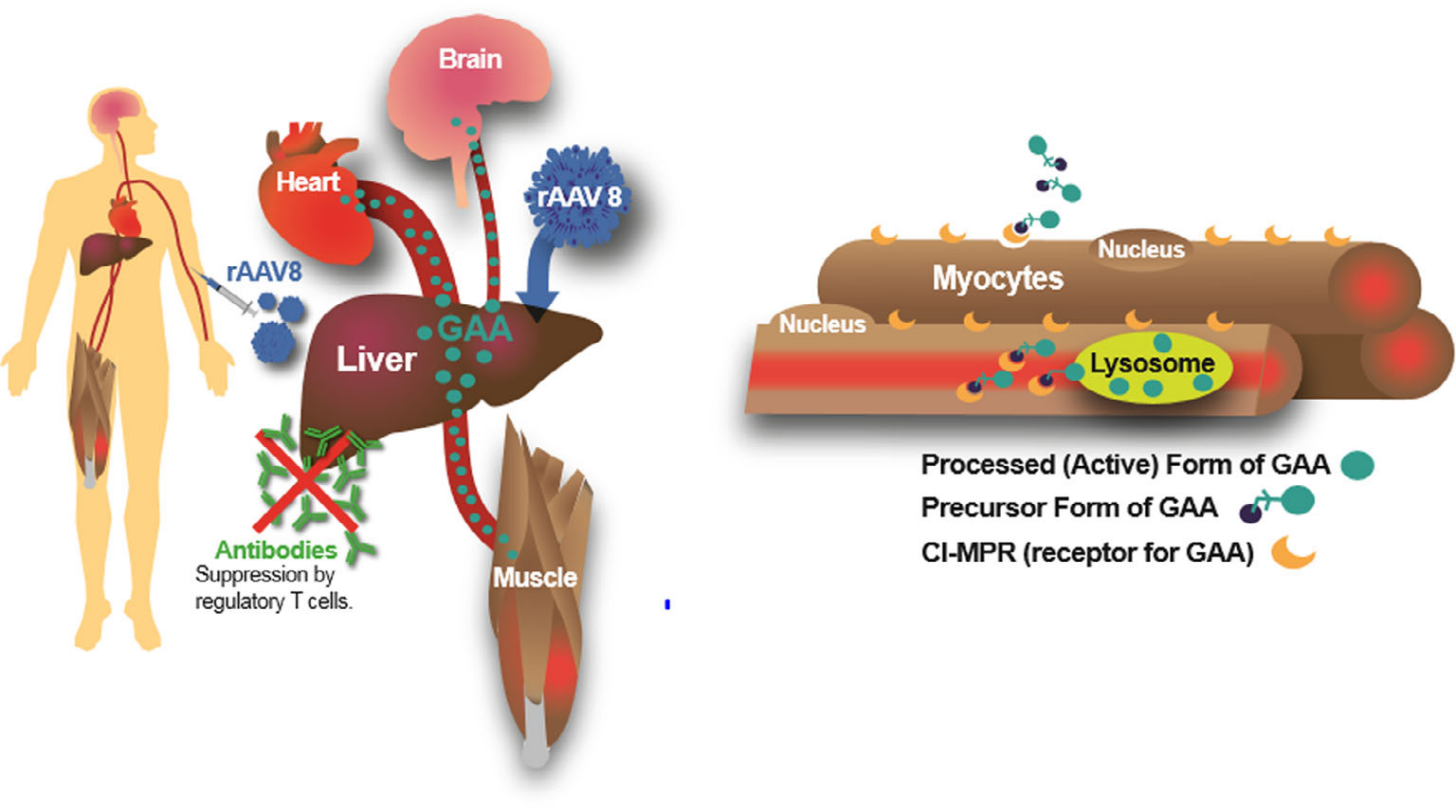
AAV2/8‐LSPhGAA gene therapy with transduction of hepatocytes. With hepatic expression of *GAA*, protein then will act in other tissues (heart, muscle, and, at high doses, the brain) following uptake into tissue and protein processing. Ultimately, processed GAA will function in the lysosome. A benefit of liver specific *GAA* expression is suppression of anti‐GAA antibodies that can limit efficacy of gene therapy.

Given the limitations of ERT therapy, other investigational therapies have been considered, such as gene therapy, substrate reduction therapy, and next generation ERTs. It has been postulated that gene therapy targeting the liver may circumvent an immune response to the *GAA* transgene.^15^ One approach is to employ a recombinant AAV8 vector containing a liver‐specific promotor (LSP) to target transduction of hepatocytes accompanied by immune tolerance to GAA (AAV2/8‐LSPhGAA). Preclinical studies revealed that hepatic expression of *GAA* will result in secretion of GAA and uptake in affected tissues such as the heart, muscle, and (at high dosages) the brain in association with improved biomarkers for Pompe disease.^16^, ^17^, ^18^ Ultimately, the uptake of secreted GAA followed by trafficking to sarcoplasmic lysosomes and subsequent processing results in increased levels of active GAA in muscle (Figure 1). A potential benefit of liver‐specific *GAA* expression is felt to be suppression of anti‐GAA antibodies that can limit efficacy of Pompe disease therapy, which depends on the activation of regulatory T cells specific to GAA.^19^ Additionally, some individuals have pre‐existing neutralizing antibodies against AAV8 from infection with wild‐type AAV that can inhibit hepatocyte transduction, an important factor to weigh when considering eligibility for clinical trial enrollment.^20^

In this study, 19 adults were screened for consideration of a phase I clinical trial of AAV2/8‐LSPhGAA‐mediated gene therapy for which the first cohort was recently described.^21^ Baseline clinical and biochemical characterization of each of the 19 individuals is provided, broadening our understanding of the phenotypic spectrum and considerations for clinical trials in LOPD. There are numerous considerations in characterizing individuals for eligibility of an AAV clinical trial for LOPD which are pertinent to novel therapeutic investigations for other inborn metabolic disorders.

## 2 METHODS

### 2.1 Study description

A total of 19 adults with LOPD were screened for eligibility in a Phase I AAV2/8‐LSPhGAA gene therapy trial in a single site study at Duke University (ClinicalTrials.gov Identifier: NCT03285126), in conjunction with the Annual United Pompe Foundation‐Duke Annual LOPD Social and Patient Meeting. The following genetic, biochemical, and clinical information was collected: *GAA* genotype, IgG AAV8 titer measured by ELISA, AAV8 neutralizing antibodies in human embryonic kidney 293 (HEK293) cells (NAB_50_), anti‐rhGAA IgG antibody titer measured by ELISA, 6MWT, urine glucose tetrasaccharide (Glc4), creatine kinase, AST, ALT, gamma‐glutamyl transferase (GGT), upright FVC, supine FVC, upright forced expiratory volume in 1 s (FEV1), and supine FEV1. The study was approved by the Duke University Institutional Review Board.

### 2.2 Eligibility criteria

Criteria for study inclusion included: (1) confirmed diagnosis of Pompe disease by blood or skin fibroblast GAA assay and two pathogenic *GAA* variants. (2) Age of 18 years or older at enrollment. (3) Capable of giving written consent. (4) Predicted FVC within the range of 30% to less than 90% in the upright position. (5) Predicted 6MWT within the range of 30% to less than 90% predicted with assistive devices permitted. (6) Receiving ERT at a stable dose for at least 104 weeks.

Criteria for study exclusion included: (1) ELISA positive for neutralizing anti‐AAV8 capsid IgG antibodies (>1:5). (2) Invasive ventilation or noninvasive ventilation required while awake and upright. (3) FVC <20% of predicted when spine. (4) Clinically relevant illness within 2 weeks of enrollment including fever >38.2°C, vomiting more than once in 24 h, seizure, or other symptom deemed contraindicative to new therapy. (5) Any condition that would interfere with participation in the study as determined by the principal investigator. (6) Pregnancy or nursing mothers. (7) Patients on a non‐standard schedule for ERT (e.g., weekly infusions as opposed to standard of care dose of infusions every 2 weeks). (8) History of hypersensitivity to levalbuterol, bitolterol, pirbuterol, terbutaline, or salmeterol which contraindicates pulmonary function testing. (9) Anti‐GAA IgG with sustained titer ≥1:12 800 for ≥6 months at time of enrollment (performed by Sanofi Genzyme Corporation, Cambridge, MA). (10) Serology consistent with exposure to HIV, serology consistent with active hepatitis B or hepatitis C infection, or any active liver disease. (11) GGT >1.2 times the upper limit of normal. (12) Active infection based on clinical symptoms. (13) Having started respiratory muscle strength training in the preceding 12 months.

### 2.3 Clinical laboratory testing

**FIGURE 2:**
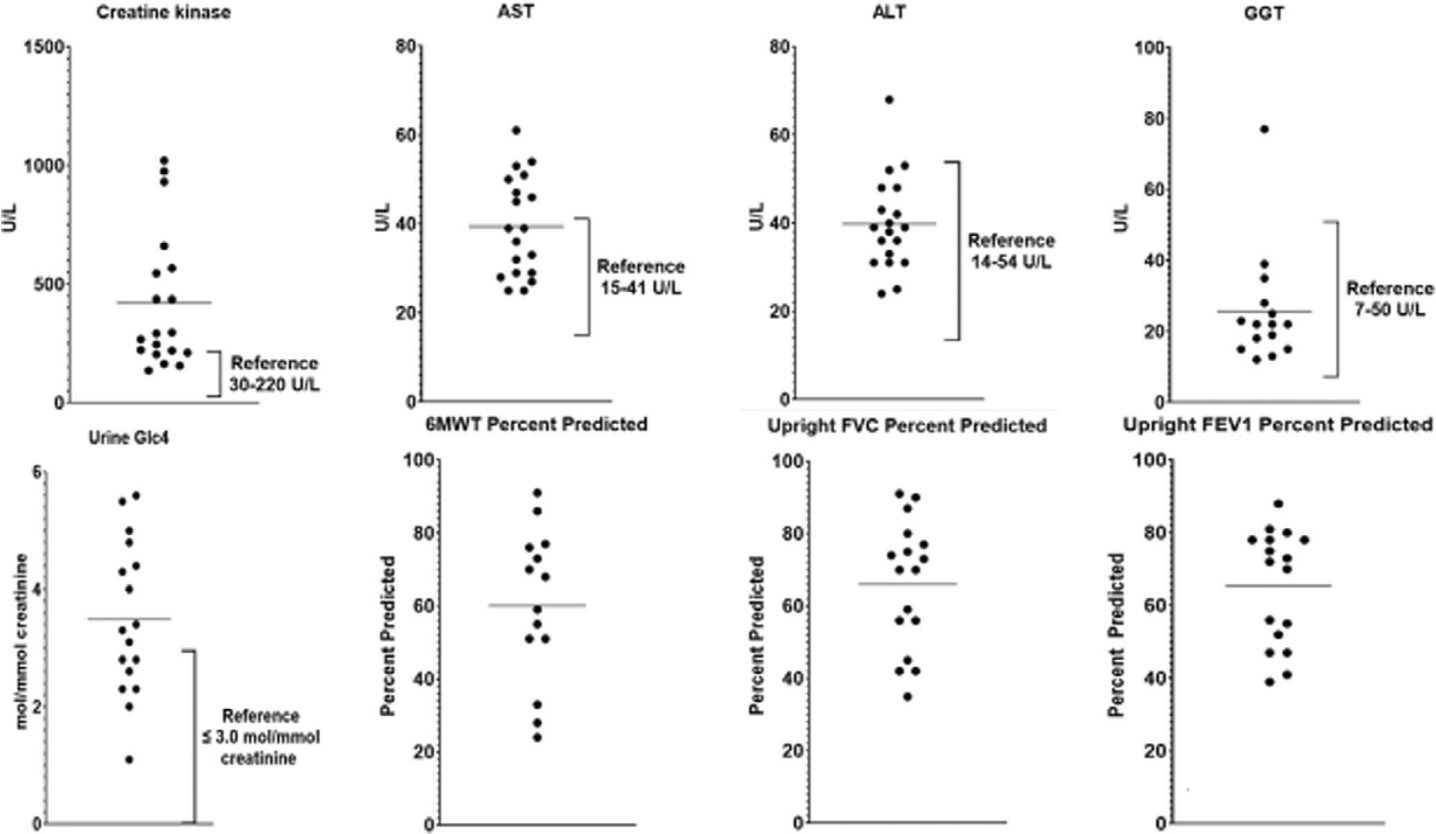
Laboratory tests, 6MWT, and upright spirometry data of study participants. Horizontal bars depict the mean value. For laboratory tests, the reference interval is depicted by brackets. 6MWT, 6‐min walk test; ALT, alanine aminotransferase; AST, aspartate aminotransferase; Cr, creatinine; FEV1, forced expiratory volume in 1 s; FVC, forced vital capacity; GGT, gamma‐glutamyl transferase; Glc4, glucose tetrasaccharide; L, liter; mol, moles; mmol, millimoles; U, unit.

Serum CK, AST, ALT, and urine Glc4 concentrations as well as anti‐rhGAA IgG antibody titers were obtained from clinical laboratory values. GAA genotyping and GAA activity levels were obtained from study records as were 6MWT and upright and supine spirometry values (Figure 2).

### 2.4 NAb assay

NAb responses to the AAV8 capsid were analyzed using a cell‐based AAV8 NAb assay described previously.^22^

## 3 RESULTS

All 19 individuals screened had the c.‐32‐13T>G *GAA* variant on one allele, a variant well described for its association with LOPD. All 19 patients had a second *GAA* variant identified (Table 1). As detailed in Table 1, there was variable expressivity in terms of laboratory values and muscle function testing (6MWT and spirometry). Eight of 19 individuals who were evaluated for study eligibility were identified to have no detectable neutralizing antibodies (Table 1) and to be eligible for the phase I gene therapy clinical trial. An additional six participants were weakly positive (titer of 1:5) for neutralizing antibodies and could be considered for enrollment.

**TABLE 1:** Clinical parameters for each of the 19 participants.

	*GAA* genotype	IgG AAV8 titer	NAb_50_	Anti‐rhGAA IgG Ab titer	6MWT (m) (% of expected distance)	Urine Glc4 (≤3.0 mmol/mol creatinine)	CK (30–220 U/L)	AST (15–41 U/L)	ALT (14–54 U/L)	GGT (7–50 U/L)	Upright FVC (L) (% pred)	Supine FVC (L)	Upright FEV1 (L) (% pred)	Supine FEV1 (L)
1	c.‐32‐13T>G; c.1447G>A	<100	5	800	161 (24%)	3.4	267	36	33	N/A	N/A	N/A	N/A	N/A
2	c.‐32‐13T>G; c.925G>A	<100	5	800	275 (55%)	2.3	546	29	40	N/A	2.37 (87%)	2.07 (76%)	1.75 (80%)	1.55 (71%)
3	c.‐32‐13T>G; c.2431delC	<100	5	400	470 (73%)	2.3	N/A	33	42	N/A	4.75 (90%)	4.11 (78%)	3.19 (78%)	2.79 (68%)
4	c.‐32‐13T>G; c.2841 + 102_2646 + 31del	400	10	1600	264 (51%)	5.5	211	39	39	N/A	2.74 (73%)	1.79 (48%)	2.09 (72%)	1.28 (44%)
5	c.‐32‐13T>G; c.1143delC	<100	<5	Negative	451 (91%)	2	292	25	31	22	N/A	N/A	N/A	N/A
6	c.‐32‐13T>G; c.1951_1952delGGinsT	<100	<5	400	425 (77%)	4	220	39	39	15	3.96 (91%)	2.84 (65%)	2.97 (88%)	2.25 (67%)
7	c.‐32‐13T>G; c.1445C>G	400	20	100	303 (51%)	4.3	255	32	25	18	1.58 (35%)	0.83 (18%)	1.43 (41%)	0.74 (21%)
8	c.‐32‐13 T > G; c.525delT	400	20	3200	381 (68%)	N/A	164	61	68	13	2.13 (59%)	1.43 (40%)	1.35 (47%)	0.81 (28%)
9	c.‐32‐13T>G; c.258dupC	<100	<5	400	557 (76%)	5	437	50	53	23	4.22 (77%)	3.64 (66%)	3.6 (81%)	2.96 (67%)
10	c.‐32‐13T>G; c.784G>A	<100	20	1600	389 (86%)	3.3	223	28	36	19	1.94 (80%)	0.83 (34%)	1.45 (75%)	0.63 (33%)
11	c.‐32‐13T>G; c.925G>A	<100	<5	12 800	312 (59%)	5.6	204	29	43	15	1.9 (56%)	1.36 (40%)	1.45 (52%)	1.00 (36%)
12	c.‐32‐13T>G; c.525delT	<100	<5	400	N/A	2.6	661	27	31	25	1.87 (56%)	1.18 (35%)	1.52 (56%)	0.90 (33%)
13	c.‐32‐13T>G; c.2481 + 102_2646 + 31del	25 600	640	800	N/A	3.1	296	47	48	35	2.72 (74%)	2.31 (63%)	2.2 (78%)	1.55 (55%)
14	c.‐32‐13T>G; c.525delT	<100	5	1600	182 (28%)	1.1	436	46	36	28	2.94 (75%)	2.30 (58%)	2.25 (70%)	1.69 (53%)
15	c.‐32‐13T>G; c.2481 + 102_2646 + 31del	<100	5	1600	150 (33%)	4.4	136	54	31	77	1.77 (42%)	N/A^a^	1.54 (47%)	N/A^a^
16	c.‐32‐13T>G; c.1124G>T	<100	5	400	NA	N/A	567	45	38	12	2.32 (70%)	1.79 (54%)	2.02 (78%)	1.62 (63%)
17	c.‐32‐13T>G; c.1402A>T	<100	<5	Negative	479 (70%)	2.8	975	53	52	22	2.26 (45%)	1.18 (23%)	2.24 (55%)	1.13 (28%)
18	c.‐32‐13T>G; c.2608C>T	<100	<5	<100	NA	4.8	1021	51	48	39	2.13 (42%)	0.88 (17%)	1.58 (39%)	0.49 (12%)
19	c.‐32‐13T>G; c.1827delC	<100	<5	Negative	NA	2.8	156	25	24	22	3.40 (70%)	1.39 (29%)	2.78 (73%)	0.99 (26%)

*Note*: IgG AAV8 titer and anti‐rhGAA IgG Ab titer assays measured by ELISA. Normal ranges, where applicable, are shown in parentheses.

Abbreviations: 6MWT, 6‐min walk test; AAV8, adeno‐associated virus serotype 8; ALT, alanine aminotransferase; AST, aspartate aminotransferase; CK, creatine kinase; Cr, creatinine; FEV1, forced expiratory volume in 1 s; FVC, forced vital capacity; GGT, gamma‐glutamyl transferase; Glc4, glucose tetrasaccharide; IgG, immunoglobulin G; L, liter; m, meters; mol, moles; mmol, millimoles; N/A, not applicable and test not performed; NAb_50_, AAV8 neutralizing antibodies in HEK293 cells; rhGAA, recombinant human GAA; U, unit; % pred, percent predicted.

^a^ Unable to tolerate supine pulmonary function testing.

The average and standard deviation calculations are also provided for FVC percent predicted positional difference, 6MWT, 6MWT percent of expected difference, urine Glc4 concentration, CK concentration, AST concentration, ALT concentration, and GGT concentration (Table 2).

**TABLE 2:** Summary of average and standard deviation of pertinent clinical parameters.

	Upright FVC percent predicted	FVC percent positional difference	6MWT (m)	6MWT percent of expected distance	Urine Glc4 (≤3.0 mmol/mol creatinine)	CK (30–220 U/L)	AST (15–41 U/L)	ALT (14–54 U/L)	GGT (7–50 U/L)
Average	66	33.2	343	60	3.5	392.6	39.4	39.8	25.7
Standard deviation	18	16.6	128	21	1.3	267.8	11.2	10.7	16.1

*Note*: Normal ranges, where applicable, are shown in parentheses.

Abbreviations: 6MWT, 6‐minute walk test; ALT, alanine aminotranferase; AST, aspartate aminotransferase; CK, creatine kinase; Cr, creatinine; FVC, forced vital capacity; GGT, gamma‐glutamyl transferase; L, liters; m, meters; mol, moles; mmol, millimoles; U, units.

No elevation of ALT was observed in this group of LOPD patients treated with ERT other than one participant with mildly increased ALT of 68 U/L (reference interval 14–54;Tables 1 and 2). GGT, an alternative biomarker for hepatitis, demonstrated a similar pattern with a single mildly increased value in a different participant of 77 U/L (reference interval 7–50; Tables 1 and 2).

## 4 DISCUSSION

The clinical disease among the 19 study participants reflects the variable expressivity of LOPD. The value of 6MWT as percent of predicted distance and positional spirometry in identifying disease progression is noted, and the normal baseline ALT is of particular importance when considering possible markers of hepatitis when administering liver‐targeted AAV gene therapy. GGT could be considered as an alternative biomarker for hepatitis that is not elevated related to muscle damage from LOPD. In this study, ALT was elevated in one individual, and GGT was elevated in another individual. Serum CK was within the normal range in both individuals, suggesting that hepatotoxicity would be the underlying source for elevated transaminases. We consider ALT at a concerning concentration when it exceeds 1.5 times the upper limit of normal, because it is used as a biomarker for T‐cell immunity against AAV.^23^ Both AST and ALT can be elevated due to muscle damage in Pompe disease, which could complicate the use of ALT as a marker of AAV‐related hepatitis to indicate the need for immunosuppression. At least for patients with LOPD enrolled in this study, prior treatment with ERT normalized ALT and therefore monitoring for hepatitis will be possible in a phase I clinical trial of gene therapy.

This trial, utilizing a study design intended to detect safety of AAV2/8‐LSPh*GAA*‐mediated gene therapy, aims for expression in the hepatocyte to leverage the secretion of GAA from a liver depot to correct GAA deficiency in skeletal and respiratory muscles to treat LOPD.^15^ Rather than peaks and troughs that occur with ERT, there should be persistent expression following delivery of gene therapy. The presence of anti‐AAV antibodies is an important consideration when determining eligibility for gene therapy trials. The percent of individuals in this study with anti‐AAV8 antibodies is comparable to a study of individuals with Factor VIII deficiency in which 23% had elevated antibodies to AAV8.^24^ This paper highlights lessons learned from the screening data for a phase I clinical trial of AAV‐mediated gene therapy collected from 19 individuals with LOPD.

It is important to acknowledge that differences in autophagic build‐up between study participants may affect the efficacy of the gene therapy. Impaired autophagy in Pompe disease has been characterized in mouse models and human specimens. It has been suggested that autophagic build‐up may affect the efficacy of ERT in Pompe disease, with some studies supporting an effect on trafficking.^25^, ^26^, ^27^, ^28^, ^29^, ^30^ Given the variable expressivity of LOPD, participants may have varying levels of autophagic build‐up which could impact results of this AAV trial as this therapeutic would also depend on effective trafficking and processing of GAA in the skeletal muscle. Further, screening of individuals for LOPD clinical trials may be limited by a lack of biomarkers for autophagic build‐up.

Thorough characterization of baseline clinical parameters is critical for both determining participant eligibility and for monitoring response to therapy. Outcomes reported in this study include functional physical therapy assessment (6MWT), disease biomarkers, and positional spirometry. It is clear that the participants screened had significant variability in clinical severity. Of note, each participant screened for this clinical trial had the c.‐32‐13T>G variant on one *GAA* allele. Variation in clinical severity could be in part due to genotype–phenotype correlation related to the other *GAA* variant, age, and presence of anti‐rhGAA antibodies, among other factors. The combination of functional physical therapy assessment, positional spirometry, and biomarkers offers valuable insight into baseline disease severity for which response to therapy can be evaluated; these three categories of clinical parameters may have pertinence for clinical trials in other Mendelian disorders of striatal muscle.

Neutralizing antibodies against AAV8 inhibit transduction of the liver and are a critical laboratory assay in determining who is more likely to experience a clinical benefit from an AAV gene therapy trial. Screening of these 19 individuals identified 8 patients with no detectable neutralizing antibodies who were eligible for the clinical trial, and an additional 6 patients who had weakly positive neutralizing antibodies (titer 1:5) who could be considered for enrollment. The high frequency of positivity for neutralizing anti‐AAV8 antibodies with titer >1:5 (26%) raises the need to consider future studies with immunosuppressive therapy, or other strategies, to deplete anti‐AAV antibodies in individuals with Pompe disease to make AAV vector‐mediated gene therapy available to them.

Determining the best parameters to measure efficacy of intervention and overcoming immune phenomena are problems that are broadly relevant as AAV vector‐mediated gene therapy will likely be developed as a new therapeutic modality for multiple inborn errors of metabolism.

## AUTHOR CONTRIBUTIONS

**William B. Hannah:** Organized data, wrote paper. **Laura E. Case:** Muscle testing; Performed research; Analyzed data; Reviewed, edited, and approved the manuscript. **Edward Smith:** Reviewed, edited, and approved the manuscript. **Deeksha Bali:** Supervised GAA activity and glycogen content measurement; Data analysis; Reviewed, edited, and approved the manuscript. **Priya S. Kishnani:** Clinical trial design; Data interpretation; Reviewed, edited, and approved the manuscript. **Dwight D. Koeberl:** Designed the study; Data interpretation; Wrote paper.

## FUNDING INFORMATION

The project described was supported by the National Center for Advancing Translational Sciences (NCATS) by NCATS Award Number UL1TR001117 to the Duke Translational Medicine Institute/Duke CTSA, and by the Alice and Y.‐T. Chen Pediatric Genetics and Genomics Center.

## CONFLICT OF INTEREST STATEMENT

Dr. Dwight D. Koeberl and Dr. Priya S. Kishnani have developed the technology that is being used in the study. If the technology is commercially successful in the future, the developers and Duke University may benefit financially. Dr. Dwight D. Koeberl has served as a consultant for Sangamo Therapeutics and for Genzyme Sanofi, Amicus, and Vertex; has received grant support from Viking Therapeutics, Genzyme Sanofi, Roivant Rare Diseases, and Amicus; and has equity in Askbio, which is developing gene therapy for Pompe disease. Dr. William B. Hannah has received consulting fees from PTC Therapeutics and ReCode Therapeutics. Dr. Edward C. Smith received salary support for his role as PI on this study. Dr. Laura E. Case has received honoraria from Genzyme Sanofi and Amicus, has participated in research supported by Genzyme Sanofi, Amicus, AskBio, Valerion, Biomarin, and by Roivant Sciences; and is a member of the Pompe Registry North American Board of Advisors Genzyme Sanofi. Dr. Deeksha Bali has received research grant support and travel funds from Genzyme Sanofi, Baebies Inc., Biomarin, Alexion Inc., SOBI biopharma, and JCR biopharma. Dr. Priya S. Kishnani has received research/grant support from Sanofi Genzyme and Amicus Therapeutics. She received consulting fees and honoraria from Sanofi Genzyme, Amicus Therapeutics, Maze Therapeutics, JCR Pharmaceutical, and Asklepios Biopharmaceutical, Inc. (AskBio). She is a member of the Pompe and Gaucher Disease Registry Advisory Board for Sanofi Gezyme, Amicus Therapeutics, and Baebies. Dr. Priya S. Kishnani has equity in Asklepios Biopharmaceutical, Inc. (AskBio) which is developing gene therapy for Pompe disease and has equity in Maze Therapeutics which is developing a small molecule therapy for Pompe disease.

## ETHICAL APPROVAL

The study was approved by the Duke University Institutional Review Board.

## Data Availability

Data archiving is not mandated, but data will be made available on reasonable request.
